# The Mechanism of Action of the Histone Deacetylase Inhibitor Vorinostat Involves Interaction with the Insulin-Like Growth Factor Signaling Pathway

**DOI:** 10.1371/journal.pone.0024468

**Published:** 2011-09-08

**Authors:** Rive Sarfstein, Ilan Bruchim, Ami Fishman, Haim Werner

**Affiliations:** 1 Department of Human Molecular Genetics and Biochemistry, Sackler School of Medicine, Tel Aviv University, Tel Aviv, Israel; 2 Gynecologic Oncology Unit, Department of Obstetrics and Gynecology, Meir Medical Center, Kfar Sava, Israel; University of Chicago, United States of America

## Abstract

A correlation between components of the insulin-like growth factor (IGF) system and endometrial cancer risk has been shown in recent studies. The antitumor action of vorinostat, a histone deacetylase inhibitor, involves changes in the expression of specific genes via acetylation of histones and transcription factors. The aim of this study was to establish whether vorinostat can modify the expression of specific genes related to the IGF-I receptor (IGF-IR) signaling pathway and revert the transformed phenotype. Human endometrioid (Type I, Ishikawa) and uterine serous papillary (Type II, USPC-2) endometrial cancer cell lines were treated with vorinostat in the presence or absence of IGF-I. Vorinostat increased IGF-IR phosphorylation, produced acetylation of histone H3, up-regulated pTEN and p21 expression, and reduced p53 and cyclin D1 levels in Ishikawa cells. Vorinostat up-regulated IGF-IR and p21 expression, produced acetylation of histone H3, and down-regulated the expression of total AKT, pTEN and cyclin D1 in USPC-2 cells. Of interest, IGF-IR activation was associated with a major elevation in IGF-IR promoter activity. In addition, vorinostat treatment induced apoptosis in both cell lines and abolished the anti-apoptotic activity of IGF-I both in the absence or presence of a humanized monoclonal IGF-IR antibody, MK-0646. Finally, vorinostat treatment led to a significant decrease in proliferation and colony forming capability in both cell lines. In summary, our studies demonstrate that vorinostat exhibits a potent apoptotic and anti-proliferative effect in both Type I and II endometrial cancer cells, thus suggesting that endometrial cancer may be therapeutically targeted by vorinostat.

## Introduction

Endometrial cancer is the most common gynecologic cancer in Western countries. The incidence of the disease has been increasing in recent years as a result of the growing obesity epidemics and, therefore, endometrial cancer constitutes a major public health issue. Endometrial cancers are classified into two major groups, Type I and Type II, with Type I being the most frequent (more than 80% of cases) [1], [2]. Type I tumors are usually estrogen-dependent, low-grade neoplasms, with an endometrioid, well-differentiated morphology, and are generally associated with a relatively good prognosis. Type II tumors appear at an advanced age, are not associated with exposure to estrogens, display a less differentiated phenotype, and have a worse prognosis. Uterine serous carcinoma (USC) constitutes the predominant histological class among Type II tumors [3], [4]. USC represents 10% of all endometrial carcinomas, is diagnosed at an advanced stage, and accounts for 50% of all relapses of the endometrial cancers, with a 5-year survival rate of 55%. A number of different genetic abnormalities have been detected in Type I endometrial cancers, including microsatellite instability and mutations of the pTEN, k-RAS, and ß-catenin genes. Type II tumors, on the other hand, often exhibit p53 mutations and loss of heterozygosity on several chromosomes [5].

The insulin-like growth factor (IGF) system plays an important role in the biology of endometrial cancer [6]. Correlations between the expression of components of the IGF system and endometrial cancer risk and development have been reported [7], [8]. Most of the biological actions of IGF-I and IGF-II are mediated by the IGF-I receptor (IGF-IR), a membrane-bound heterotetramer with potent anti-apoptotic and cell-survival activities [9]–[11]. The IGF-IR emerged in recent years as a promising molecular target in oncology and a number of approaches are currently being employed to target the receptor for therapeutic purposes [8], [12].

Vorinostat is a novel histone deacetylase inhibitor (HDAC) (Zolinza, suberoylanilide hydroxamic acid or SAHA; Merck Research Laboratories), representing a new class of potential antitumor agents. Vorinostat induces growth arrest, differentiation, and/or apoptosis in a variety of transformed cells, including prostate, leukemia, breast, and colon cancers [13], and has undergone initial evaluation in Phase I and II clinical trials [14], [15]. The mechanism/s underlying the anti-tumor action of vorinostat is not yet clear but may involve changes in the expression of specific genes *via* acetylation of histones and transcription factors as well as non-transcriptional effects such as inhibition of mitosis. The aim of this study was to establish whether the mechanism of action of vorinostat involves changes in the expression or activity of specific genes related to the IGF-IR signaling pathway. In addition, we aimed at characterizing the anti-carcinogenic and pro-apoptotic actions of vorinostat in both Type I and II endometrial cancer cells, including its potential ability to render the cells more sensitive to chemotherapy.

## Materials and Methods

### Cell lines and treatments

The human endometrioid Ishikawa cell line (Type I) was obtained from Dr. Y. Sharoni, Ben Gurion University, Beer-Sheba, Israel. Uterine serous papillary (USPC-1 and USPC-2; Type II) endometrial cancer cell lines were kindly provided by Dr. A. Santin, Yale University School of Medicine, New Haven, CT, USA. Ishikawa cells were grown in Dulbecco's modified Eagle's medium (DMEM) (Gibco BRL®, Paisley, Scotland) and USPC cells were grown in RPMI-1640 medium (Biological Industries Ltd., Kibbutz Beit-Haemek, Israel). Both media were supplemented with 10% fetal bovine serum (FBS), 2 mM glutamine, and 50 µg/ml gentamicin sulfate. All reagents were purchased from Biological Industries Ltd. In addition, 5.6 mg/l amphotericin B was added (Sigma-Aldrich, St. Louis, MO, USA). Cells were incubated at 37°C in a humidified atmosphere containing 5% CO_2._ Vorinostat was obtained from Merck, Sharp & Dohme Israel (Petach Tikva, Israel), kept as a stock solution (100 mM) in DMSO and stored at −20°C. In all of the experiments, cells were serum-starved for 24 h, after which they were treated with 5 µM vorinostat, in the absence or presence of IGF-I (50 ng/ml) (PeproTech Ltd, Rocky Hill, NJ, USA). For cell growth assays, doses of 3 µM or 5 µM vorinostat were used, either in the absence or presence of 5 µM or 10 µM cisplatin (Sigma-Aldrich). In some experiments, cells were treated with 5 µM vorinostat for 24 h, separately and in combination with 10 µg/ml MK-0646, a humanized monoclonal IGF-IR antibody (Merck & Co Inc., Whitehouse Station, NJ, USA) during the last 5 h of the incubation period, or in the presence or absence of IGF-I (50 ng/ml) for 24 h, or during the last 10 min of the incubation period, as indicated.

### Western immunoblots

Cells were lysed in a buffer containing protease inhibitors (#9803, Cell Signaling Technology Beverly, MA, USA). Protein content was determined using the Bradford reagent (Bio-Rad Ltd., Hercules, CA, USA) and bovine serum albumin (BSA) as a standard. Samples were electrophoresed through 15%, 12%, 10%, 7.5% or 5% SDS-PAGE gels, followed by blotting of the proteins onto nitrocellulose membranes. After blocking with either skim milk and/or 3% BSA, the blots were incubated overnight with the antibodies listed below, washed, and incubated with the appropriate horseradish peroxidase (HRP)-conjugated secondary antibody. Antibodies against phospho-IGF-IR (3024), IGF-IR β-subunit (3027), insulin receptor [(IR); 3025], phospho-AKT (9271), AKT (9272), phospho-ERK1/2 (9106), poly ADP ribose polymerase [(PARP); 9542], caspase 3 (9661), phospho-Ampk (2531), Ampk (2532), phospho-mTor (5536), mTor (2983), and PI3K p85 (4292) were obtained from Cell Signaling Technology. Antibodies against ERK1 (K-23), p21 (C-19), Bcl2 (C-2), p53 (mixture of DO-1 and Pab 1801), pTEN (A2B1), cyclin D1 (H295), and caspase 9 (H-83) were purchased from Santa Cruz Biotechnology (Santa Cruz, CA, USA). An antibody against actin (Clone C4) was purchased from ICN Biomedicals, Inc. (Aurora, OH USA), anti-acetyl histone H3 was from Upstate/Millipore (Billerica, MA, USA), and anti-caspase 8 (M032-3) was obtained from Medical & Biological Laboratories Co. Ltd. (Wobum, MA, USA). The secondary antibodies were HRP-conjugated goat anti-rabbit IgG (1∶50,000) and donkey anti-mouse IgG (1∶25,000; Jackson ImmunoResearch Laboratories, West Grove, PA, USA). Proteins were detected using the SuperSignal West PicoChemiluminescent Substrate (Pierce, Rockford, IL, USA). The expression of actin was measured as a loading control of total proteins.

### Transfections and luciferase assays

For transient cotransfection experiments an IGF-IR promoter luciferase reporter construct including 476 bp of the 5′-flanking and 640 bp of the 5′-untranslated regions of the IGF-IR gene [p(−476/+640) luciferase (LUC)] was employed [16], [17]. Ishikawa and USPC-2 cells were seeded in six-well plates the day before transfection and cotransfected with 1 µg of the IGF-IR promoter reporter along with 0.2 µg of a ß-galactosidase plasmid (pCMVß, Clontech, Palo Alto, CA, USA), using the Jet-PEI transfection reagent (Polyplus, Illkirch, France). Vorinostat was added to the medium after 24 h. Cells were harvested 48 h after transfection and luciferase and ß-galactosidase activities were measured as described [16]. Promoter activities were expressed as luciferase values normalized for ß-galactosidase activity.

### Proliferation assays

Cells were seeded onto 24-well plates (1.8×10^4^ Ishikawa cells/well and 3.6×10^4^ USPC-2 cells/well). After 24 h, the cells were treated with vorinostat in combination with cisplatin for 24 h, 48 h, or 72 h in triplicate wells. Cell viability was assessed using a standard Thiazolyl Blue Tetrazolium Bromide (MTT) method [18]. The color developed was quantitated by measuring absorbance at a wavelength of 530 nm and reference wavelength of 630 nm on a Microplate Reader (SpectraMax 190, Molecular Devices, Sunnyvale, CA, USA). Cell viability was expressed as percentage of optical density values obtained upon treatment relative to control.

### Cell cycle analysis

Cells were seeded in 6-well plates (1×10^6^ cells/well) for 24 h. Cells were then serum-starved for an additional 24 h and incubated in the presence or absence of vorinostat for 72 h. After incubation cells were washed with phosphate buffered saline, trypsinized, permeabilized with Triton X-100 (4%) and stained with propidium iodide (50 µg/ml). Stained cells were analyzed using a FacsCalibur system (Cytek Development Inc, Fremont, CA, USA).

### Soft agar colony formation assays

To determine colony formation in soft agar, cells were added to the top-layer agar (Difco Noble agar, Difco Laboratories, Detroit, MI, USA), in triplicate [19]. Briefly, 2 ml of 0.6% soft agar/complete culture medium at 42°C were added to Ishikawa (2.5×10^4^ Ishikawa cells/well) and USPC-1 (1.2×10^4^ USPC-1 cells/well) cells, gently mixed by pipetting up and down, and put onto 6-well plates with a 0.6% soft agar underlay. The agar was allowed to harden for 10 min and then incubated at 37°C. Vorinostat was added 24 h after platting, and the cells were incubated for another 22 days. The medium overlay was changed twice a week. Colonies were photographed with 40× and 200× objectives and the number of colonies larger than 3 mm diameter was counted using an inverted microscope.

### Wound-healing assays

A wound-healing assay was performed using a previously described protocol [20]. Briefly, Ishikawa and USPC-2 cells were grown to confluence in 6-well plates, and a wound was made in the cell monolayer using a sterile micropipette tip. Then, cells were cultured at 37°C in starvation medium, in the presence or absence of IGF-I in combination with vorinostat. At time 0, the scratched monolayer cultures were photographed using an inverted microscope (ECLIPSE Ti, Nikon Corp, USA). Cell movement was assessed 48 h and 72 h after wounding from photographs taken under the microscope with a 10× objective.

## Results

### Effect of vorinostat on the IGF-I signaling pathway

**Figure 1:**
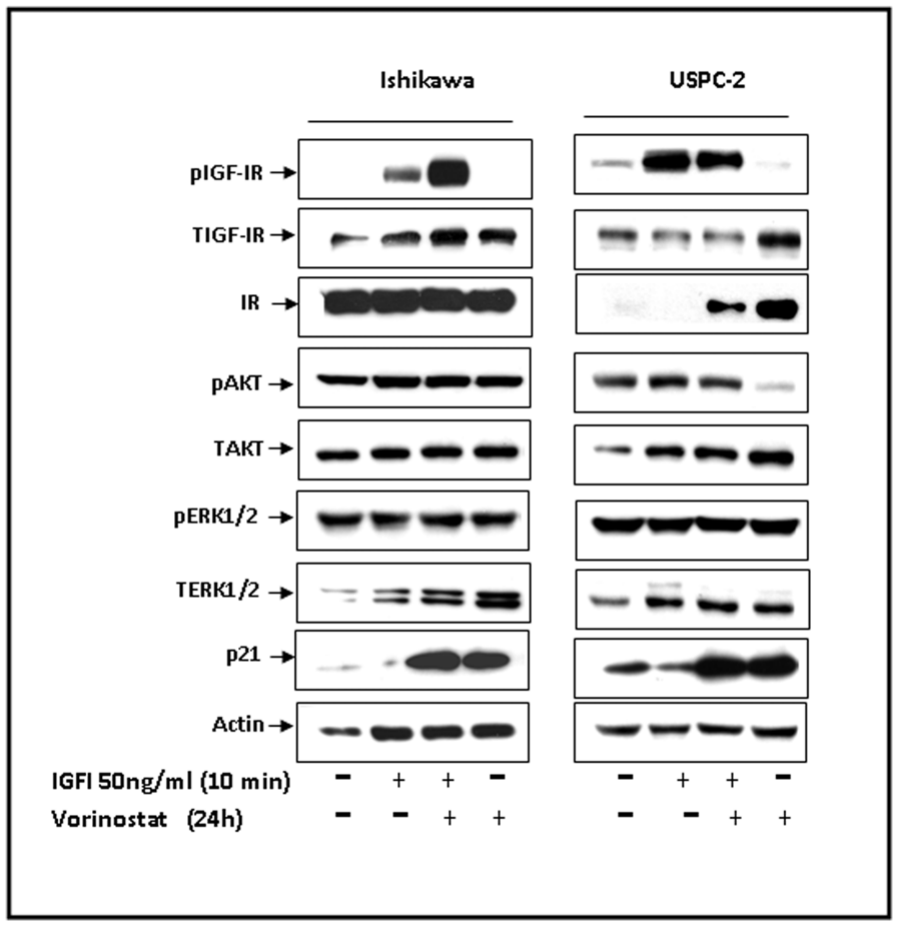
Effect of vorinostat on IGF-I-mediated signal transduction. Ishikawa and USPC-2 cells were treated for 24 h with vorinostat (or left untreated) and/or IGF-I during the last 10 min of the incubation period. Whole-cell lysates (100 µg) were resolved on SDS-PAGE and immunoblotted with antibodies against pIGF-IR, TIGF-IR, pAKT, TAKT, pERK1/2, TERK1/2, p21, and actin. The figure shows the result of a typical experiment, repeated three times with similar results. IGF-IR phosphorylation in USPC-2 cells was significantly lower than in Ishikawa cells and was detected by Western blot analysis only after longer exposure times of blots to X-ray film. Therefore, this experiment does not allow comparison between both cell lines in terms of levels of expression or phosphorylation.

To evaluate the potential regulation of the expression and activation of IGF-IR and downstream signaling mediators by vorinostat in endometrial cancer, Ishikawa and USPC-2 cells were treated with vorinostat for 24 h, in the presence or absence of IGF-I during the last 10 min of the incubation period (Figure 1). As expected, IGF-I stimulated phosphorylation of IGF-IR in both cell lines. Vorinostat enhanced the IGF-I-stimulated IGF-IR phosphorylation in Ishikawa cells. In contrast, pretreatment with vorinostat slightly reduced the IGF-I-induced phosphorylation of IGF-IR in USPC-2 cells. Vorinostat alone induced a moderate decrease in IGF-IR phosphorylation compared with untreated cells. Vorinostat markedly up-regulated the level of p21 in both cell types. The induction of p21 is commonly used as a marker of HDAC inhibition [21]. Moreover, vorinostat up-regulated the expression of IGF-IR, insulin receptor (IR), and AKT but did not alter the expression of ERK1/2 in USPC-2 cells. In addition, vorinostat led to a decrease in AKT phosphorylation in USPC-2 cells. As shown in Figure 1, the levels of IR and AKT were similar in vorinostat-treated and untreated Ishikawa cells.

### Effect of vorinostat on the induction of apoptosis and acetyl-histone H3

**Figure 2:**
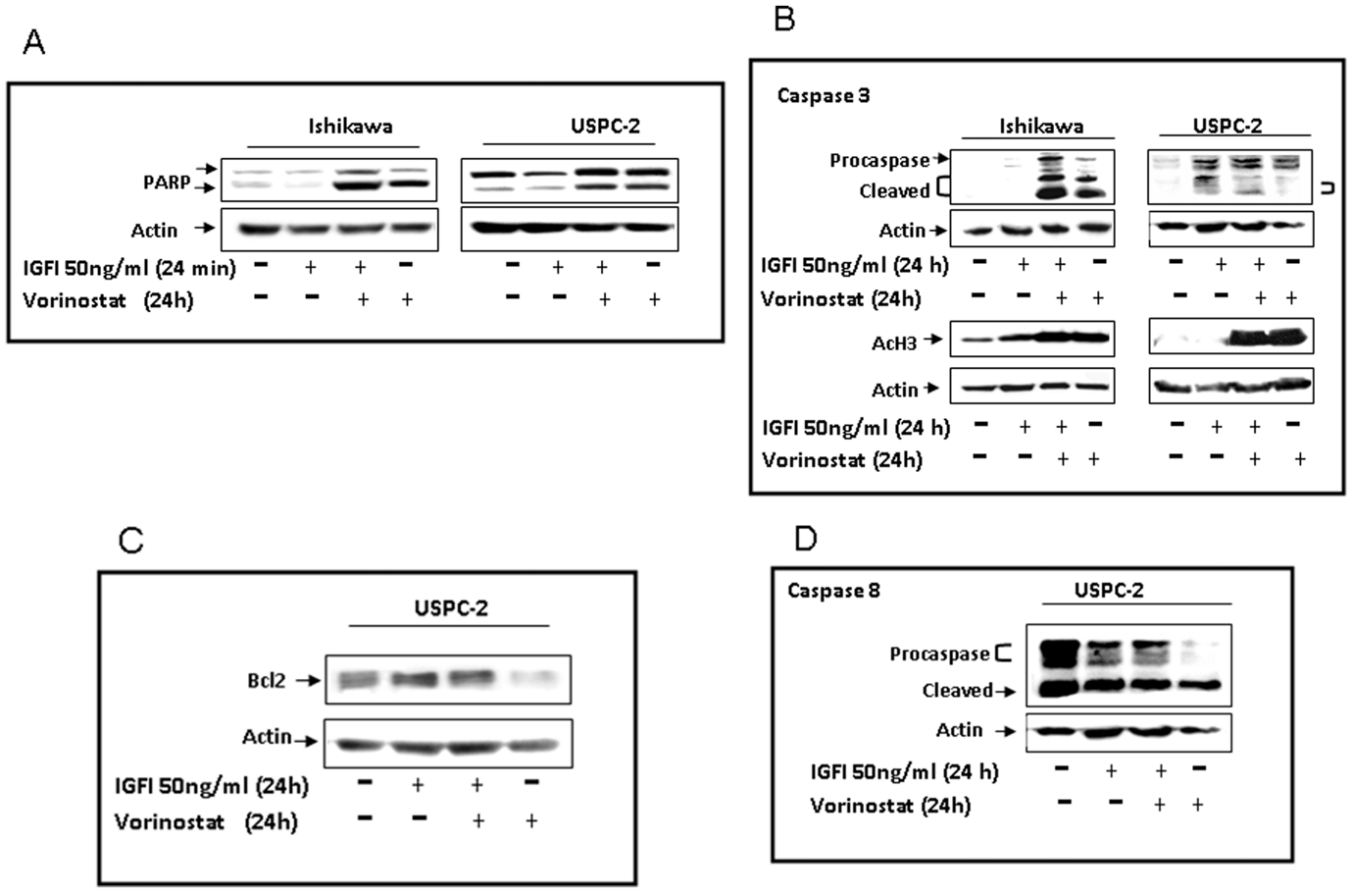
Effect of vorinostat on apoptosis and acetyl-histone H3. Western blot analysis of PARP (A) on Ishikawa and USPC-2 cells and caspase 3 (B), Bcl2 (C) and caspase 8 (D) on USPC-2 cells. Cells were treated with vorinostat for 24 h in the absence or presence of IGF-I. After incubation, cell lysates were prepared and electrophoresed (100 µg for Bcl2 or 70 µg for caspases 3 and 8). Caspase 3 expression in USPC-2 cells was detected after a long exposure time of blots to X-ray film. Results are representative of three independent experiments.

To examine the potential effect of vorinostat on apoptosis, cells were serum-starved for 24 h and then treated with vorinostat in the absence or presence of IGF-I (50 ng/ml) for 24 h. Apoptosis was assessed by cleaved PARP and caspase 3 measurements using Western blots. The cleavage of the ∼116-kDa PARP molecule into an ∼85-kDa band is considered a typical hallmark of early apoptosis [22]. Results obtained showed that, after 24 h, IGF-I treatment prevented the appearance of the ∼85-kDa band in Ishikawa cells (Figure 2A). Under our experimental conditions, however, IGF-I, by itself, had a reduced anti-apoptotic effect in USPC-2 cells. Vorinostat induced apoptosis in both cell lines and abolished the anti-apoptotic activity of IGF-I. The extent of the vorinostat-induced apoptosis, as reflected by the intensity of the ∼85-kDa PARP band, was more pronounced in Ishikawa than in USPC-2 cells. To address the potentially different sensitivity to vorinostat in both cell lines, the role of caspase 3 in regulating vorinostat-induced apoptosis was investigated. Caspase 3 has been shown to be a key component of the apoptotic machinery. Caspase 3 is activated in apoptotic cells and cleaves several cellular proteins, including PARP. Western blot analysis revealed that Ishikawa and USPC-2 cells express very low levels of endogenous caspase 3 (Figure 2B). Vorinostat led to a modest increase in the cleavage of caspase 3 in USPC-2 and to a very large increase in the cleaved form of caspase 3 in Ishikawa cells. To further explore the molecular mechanisms that control apoptosis in USPC-2 cells, Bcl2 and caspase 8 immunoblotting analyses were carried out in vorinostat-treated cells. Results obtained showed that basal Bcl2 levels were very low. Furthermore, IGF-I, both in the absence or presence of vorinostat, up-regulated, and vorinostat down-regulated Bcl2 levels (Figure 2C). Vorinostat-treated cells exhibited markedly diminished full-length caspase 8 levels. On the other hand, IGF-I treatment (both in the presence or absence of vorinostat) had a minor effect on the levels of the full-length and a cleaved form of caspase 8 with a MW of ∼25-kDa (Figure 2D). Taken together, the data indicate that vorinostat induced caspase 3 activation and PARP cleavage in Ishikawa cells. In addition, vorinostat led to a large decrease in the expression of Bcl2 and full-length caspase 8, and increased both full-length and cleaved PARP in USPC-2 cells. Finally, analysis of the acetylation level of histone H3 by Western blots showed that vorinostat hyperacetylated histone H3 in both cell lines (Figure 2B).

### Effect of vorinostat on specific transcription factors involved in IGF-IR gene regulation

**Figure 3:**
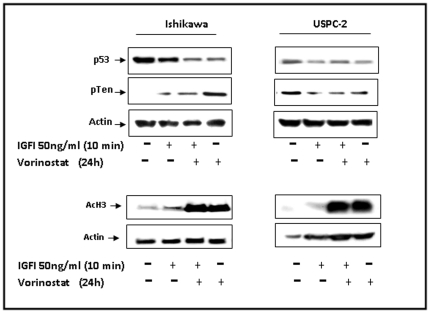
Effect of vorinostat on p53 and pTEN expression. Ishikawa and USPC-2 cells were treated for 24 h with vorinostat (or left untreated) and/or IGF-I. Whole-cell lysates (100 µg) were resolved on SDS-PAGE and immunoblotted with antibodies against p53, pTEN, acetyl-histone H3 and actin. Western blot analysis revealed that basal p53 levels were much lower in USPC-2 than in Ishikawa cells and were detected after long exposure times. Results represent a typical experiment out of three independent experiments.

Studies have shown that vorinostat modulates the transcription of multiple genes in cancer cells [23], [24]. No studies, however, have thus far addressed the effect of vorinostat on transcription factors and transcriptional mechanisms involved in regulation of IGF-IR gene expression. We chose to examine the effect of vorinostat on tumor suppressors p53 and pTEN expression. The rationale for this choice is the fact that both p53 and pTEN were shown to downregulate IGF-IR expression [25], [26]. For this purpose, cells were treated with vorinostat in the presence or absence of IGF-I, and p53 and pTEN expression was analyzed by Western blots. The data demonstrate that vorinostat increased pTEN expression and reduced p53 levels in Ishikawa cells (Figure 3). Furthermore, vorinostat down-regulated the expression of both p53 and pTEN in USPC-2 cells. Similar results were obtained when Ishikawa and USPC-2 cells were treated with vorinostat followed by IGF-I stimulation. As expected, vorinostat hyperacetylated histone H3 in both cell lines, both in the absence or presence of IGF-I (Figure 3, bottom panel).

### Effect of vorinostat on IGF-IR promoter activity

**Figure 4:**
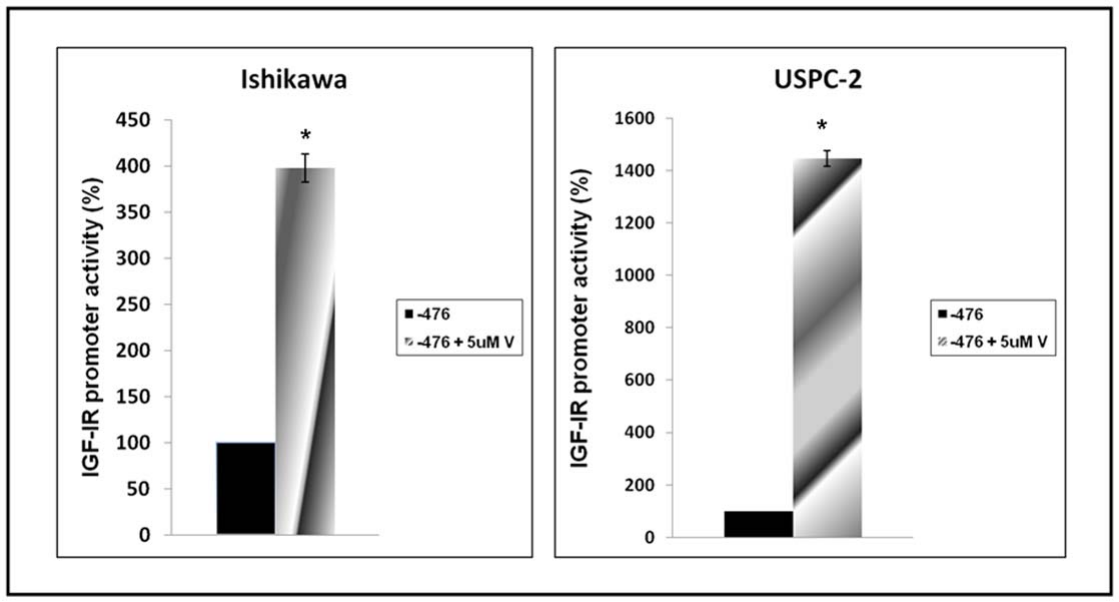
Regulation of IGF-IR promoter activity by vorinostat in endometrial cancer cells. Ishikawa and USPC-2 cells were transiently transfected with an IGF-IR promoter-luciferase reporter plasmid, p(−476/+640)LUC, which contains most of the proximal region of the IGF-IR promoter, and a ß-galactosidase vector. Promoter activity is expressed as luciferase values normalized for ß-galactosidase. A value of 100% was given to the promoter activity in the absence of vorinostat. Results are mean ± S.E.M. (three independent experiments); p*<0.01 *versus* untreated cells.

To determine whether the increase in IGF-IR gene expression in vorinostat-treated Ishikawa and USPC-2 cells is mediated at the level of transcription, cells were transiently transfected with a luciferase reporter gene under the control of the IGF-IR promoter. Twenty-four hours after transfection, vorinostat (5 µM, or vehicle) was added to the medium and incubated for an additional 24 h. Cells were then collected and promoter activity was measured. The results indicate that vorinostat induced a significant increase in IGF-IR promoter activity in both cell lines. Specifically, vorinostat enhanced IGF-IR-promoter activity by 398±15% in Ishikawa and by 1446±29% in USPC-2 cells versus control untreated cells [100% (Figure 4)].

### Effect of vorinostat and/or cisplatin on cell growth

**Figure 5:**
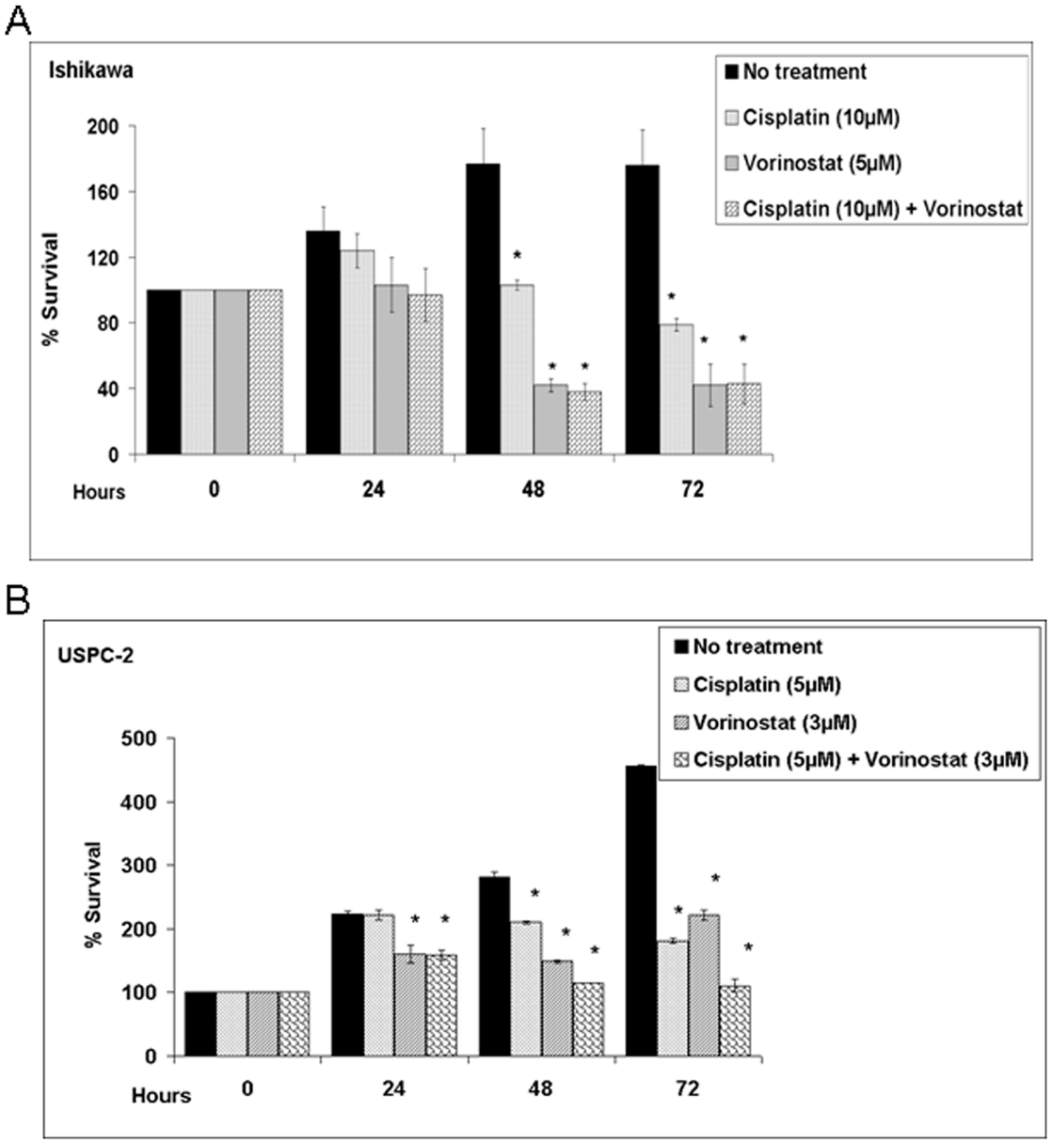
Effect of vorinostat on endometrial cancer cells proliferation. Cells were plated in 24-well plates at a density of 2×10^4^ cells/well for Ishikawa (A) and 3×10^4^ cells/well for USPC-2 (B). The bars represent the mean ± S.E.M. of three independent experiments, performed each in triplicate; * p<0.05 *versus* untreated cells.

To assess the anti-proliferative effect of vorinostat, Ishikawa and USPC-2 cells were cultured in 10% serum-containing media with vorinostat and/or cisplatin for 72 h. The proliferation rate was determined by MTT assays. Results obtained showed that vorinostat or cisplatin alone inhibited the proliferation rate in both cell lines compared with untreated cells. Thus, in Ishikawa cells, 5 µM vorinostat decreased proliferation rate by 25%, 76%, and 73% and 10 µM cisplatin decreased proliferation rate by 8.9%, 42%, and 55%, at 24 h, 48 h and 72 h, respectively (Figure 5A). Vorinostat in combination with cisplatin had no additive effect in comparison to vorinostat alone in Ishikawa cells. Marked decreases in proliferation were seen in USPC-2 cells with low doses (3 µM) of vorinostat (28%, 47%, and 51% reductions, at 24 h, 48 h, and 72 h, respectively) and cisplatin (5 µM) (5%, 25%, and 60% reductions, at 24 h, 48 h, and 72 h respectively) (Figure 5B). The combination of vorinostat with cisplatin led to significantly greater growth inhibition rates than chemotherapy alone in USPC-2 cells at 48 h and 72 h (60% and 76% reductions, respectively). These data indicate that combination treatment in these cells resulted in a synergistic decrease in proliferation compared to single agent treatment.

### Effect of vorinostat on cell cycle

To examine the effect of vorinostat on cell cycle progression, cells were incubated with vorinostat (or left untreated), stained with propidium iodide, and analyzed by flow cytometry. In Ishikawa cells, vorinostat reduced the proportion of cells at the Go/G1 phase from 60.14±1.1% to 56.96±1.9% and at the S phase from 16.31±2.5% to 3.83±0.98%, and increased the proportion of cells at the G2 phase from 13.95±1.5% to 37.33±2.2% (Table 1). Similarly, in USPC-2 cells vorinostat reduced the proportion of cells at Go/G1 from 70.79±3.6% to 49.36±2.0% and at S phase from 4.46±2.5% to 0.71±0.09%, and increased the proportion of cells at G2 from 24.44±1.5% to 48.9±1.7%.

**Table 1:** Effect of vorinostat on the cell cycle in endometrial cancer cells.

Cells	Control	Vorinostat
**Ishikawa**		
G_0_/G1	60.145±1.1	56.965±1.9*
S	16.31±2.5	3.83±0.98*
G_2_	13.95±1.5	37.335±2.2*
**USPC-2**		
G_0_/G1	70.795±3.6	49.365±2.0*
S	4.465±2.5	0.71±0.09*
G_2_	24.445±1.5	48.9±1.7*

Ishikawa and USPC-2 cells were seeded in duplicate dishes, serum-starved for 24 h, and treated with vorinostat (or left untreated, controls) for 72 h. Cell cycle distribution was assessed by FACS analysis. The values in the Table denote mean ± SEM;

*p≤0.05 *versus* controls.

### Effect of vorinostat on the mTor and Ampk pathway

**Figure 6:**
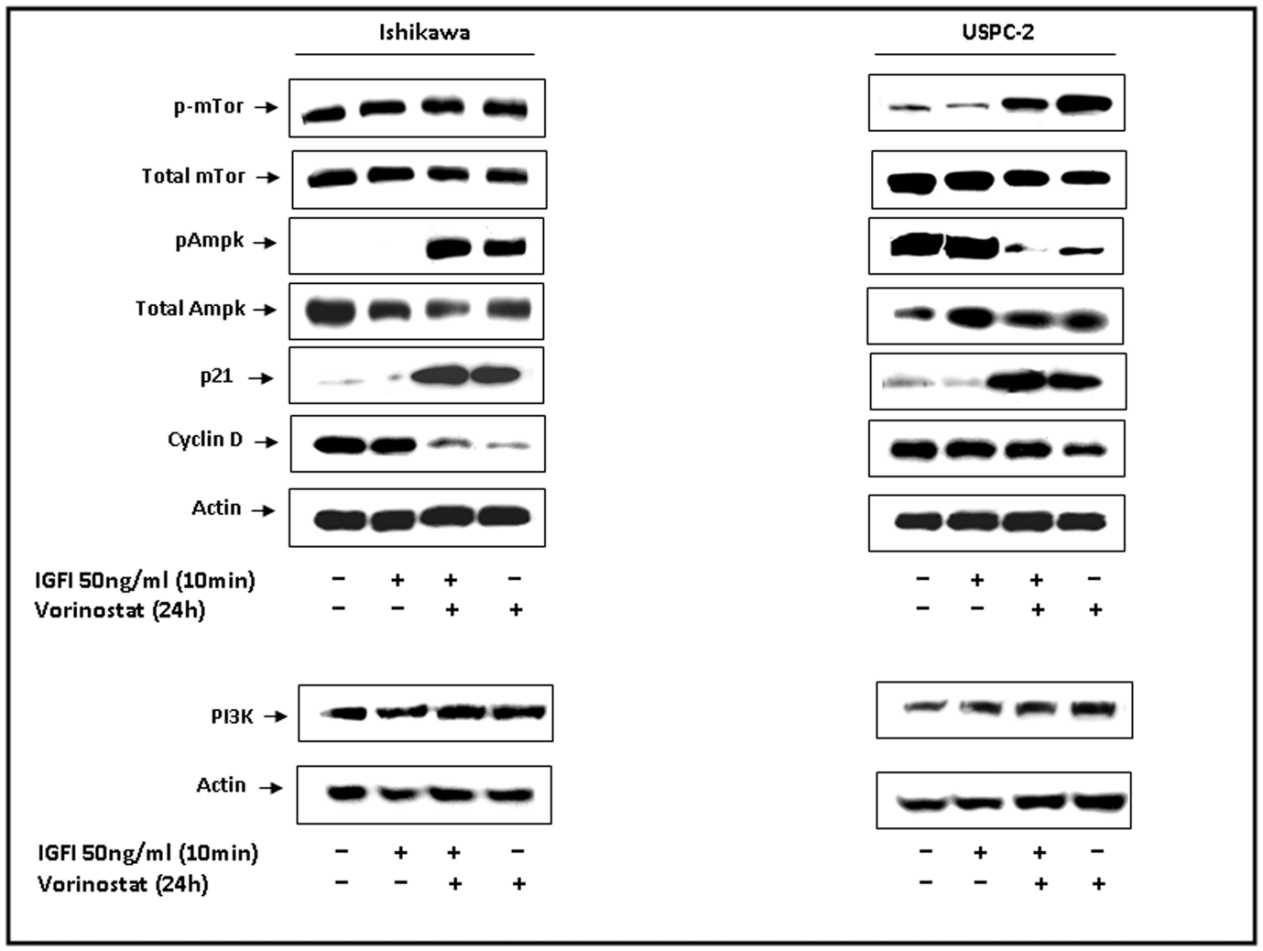
Effect of vorinostat on the mTor and Ampk signaling pathway in endometrial cancer cells. Ishikawa and USPC-2 cells were treated with vorinostat and/or IGF-I, and cell lysates (100 µg) were prepared after 24 h. Western blot analysis was performed with antibodies against pmTor, T-mTor, p-Ampk, T-Ampk, p21, cyclin D1, PI3K and actin. The figure shows the results of a typical experiment, repeated three times with similar results.

To evaluate the effect of vorinostat on the mammalian Target of Rapamycin (mTor) pathway, cells were treated with vorinostat in the presence or absence of IGF-I, and mTor and PI3K levels were measured by Western blots. Results obtained showed no changes in phosphorylation and total mTor levels after treatment with IGF-I in the absence or presence of vorinostat in Ishikawa cells (Figure 6). On the other hand, vorinostat, in the presence or absence of IGF-I, stimulated mTor phosphorylation in USPC-2 cells. No changes in total mTor levels and a mild increase in PI3K levels were seen in both cells. Next, the effect of vorinostat on Ampk was assessed. Vorinostat, in the presence or absence of IGF-I, activated Ampk in Ishikawa and reduced Ampk phosphorylation in USPC-2 cells. Cell cycle regulatory proteins p21 and cyclin D1 were also analyzed by Western blots following exposure to vorinostat. As shown above, vorinostat markedly up-regulated p21 (Figures 1 and 6) and down-regulated the expression of cyclin D1 (Figure 6) in both cell lines. This data suggest that vorinostat may cause growth arrest at the G1-S stage of the cell cycle by regulating p21 and cyclin D1 levels.

### Vorinostat inhibits colony formation in endometrial cancer cell lines

**Figure 7:**
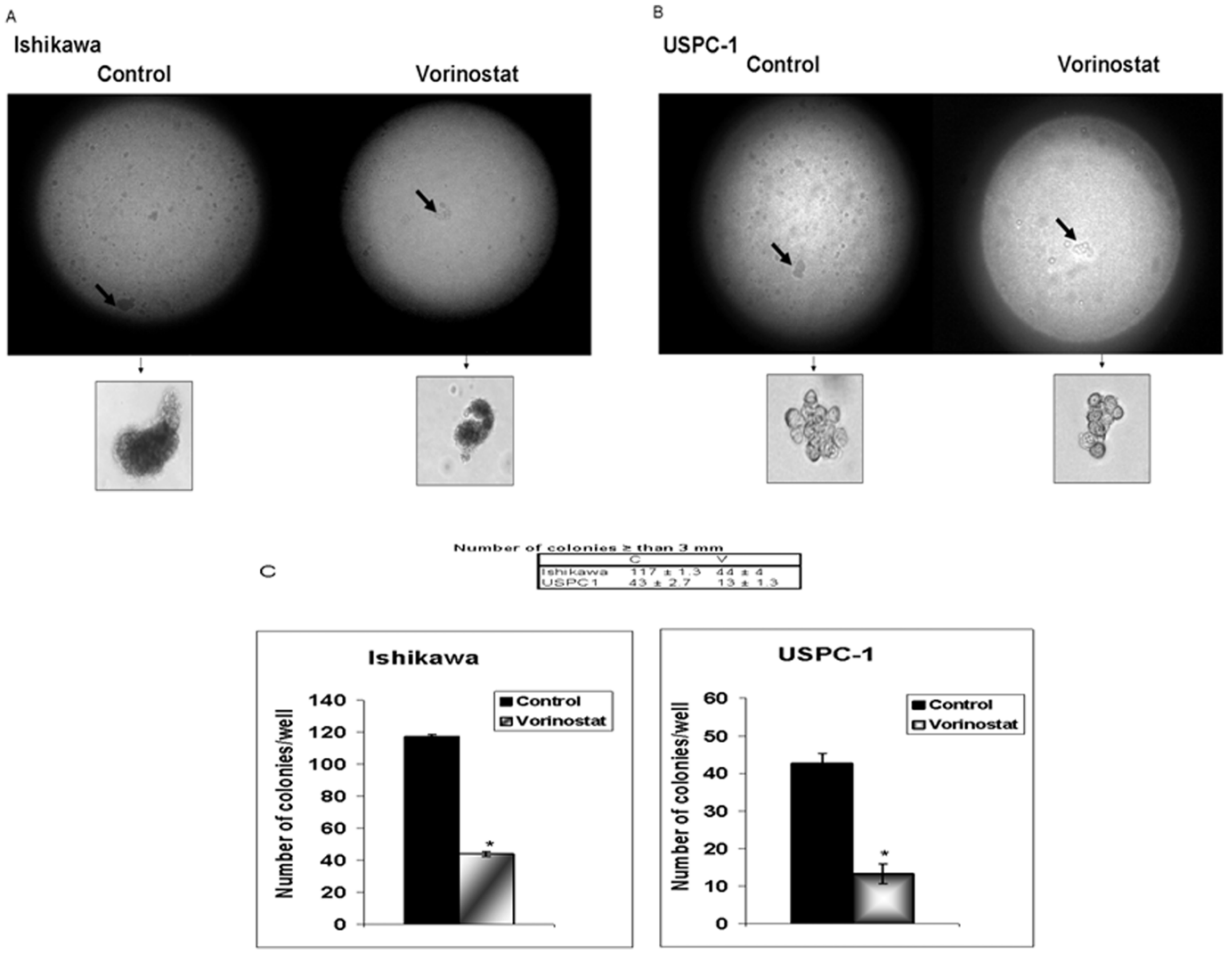
Colony formation in soft agar. A total of 1.5×10^4^ Ishikawa cells and 1.2×10^4^ USPC-1 cells were seeded on 6-well plates in soft agar. The formed colonies were photographed at a magnification of 40× and 200× (A) and colonies with diameters greater than 3 mm were counted (B) at day 22. The bars represent the mean ± S.E.M. of three independent wells; * p<0.05 *versus* untreated cells.

To corroborate the growth inhibitory effect of vorinostat, a soft-agar colony formation assay was used. Vorinostat (3 µM) led to a significant inhibition in the number of colonies in Ishikawa and USPC-1 cells (Figures 7A, B and C) compared with untreated cells (44±4 *versus* 117±1.3 colonies in Ishikawa cells, and 13±1.3 *versus* 43±2.7 colonies in USPC-1 cells).

### Vorinostat inhibits cell migration in endometrial cancer cell lines

**Figure 8:**
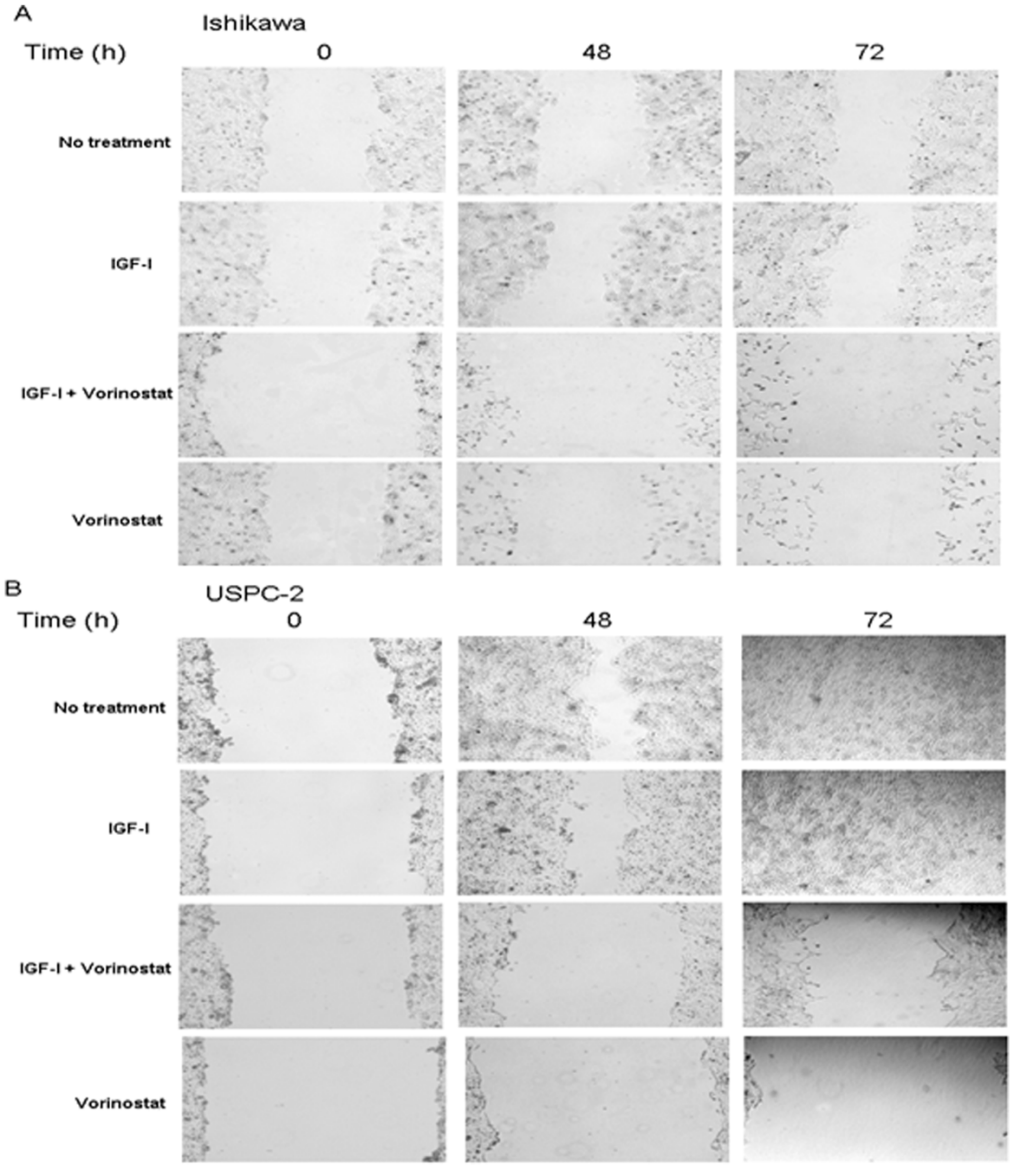
Effect of vorinostat on wound healing assays in endometrial cancer cells. Wounds were made on monolayers of Ishikawa (A) and USPC-2 (B) cells grown to 100% confluence. Untreated (control) cells or cells treated with vorinostat and/or IGF-I were photographed just after scratch (time 0), and after 48 h and 72 h. Results presented here are representative of triplicate independent samples of each cell line.

To test the effect of vorinostat on cell migration, scratch wound migration assays were performed as described in Materials and Methods. Cells were incubated in serum-free media containing IGF-I (50 ng/ml), vorinostat (5 µM), or both, for 48 h or 72 h. Data obtained showed that both untreated and IGF-I-treated Ishikawa and USPC-2 cells migrated at similar rates over 72 h (Figure 8 A and B). Vorinostat, both in the presence or absence of IGF-I, inhibited migration of both cell lines.

### Does the pro-apoptotic effect of vorinostat require IGF-IR action?

**Figure 9:**
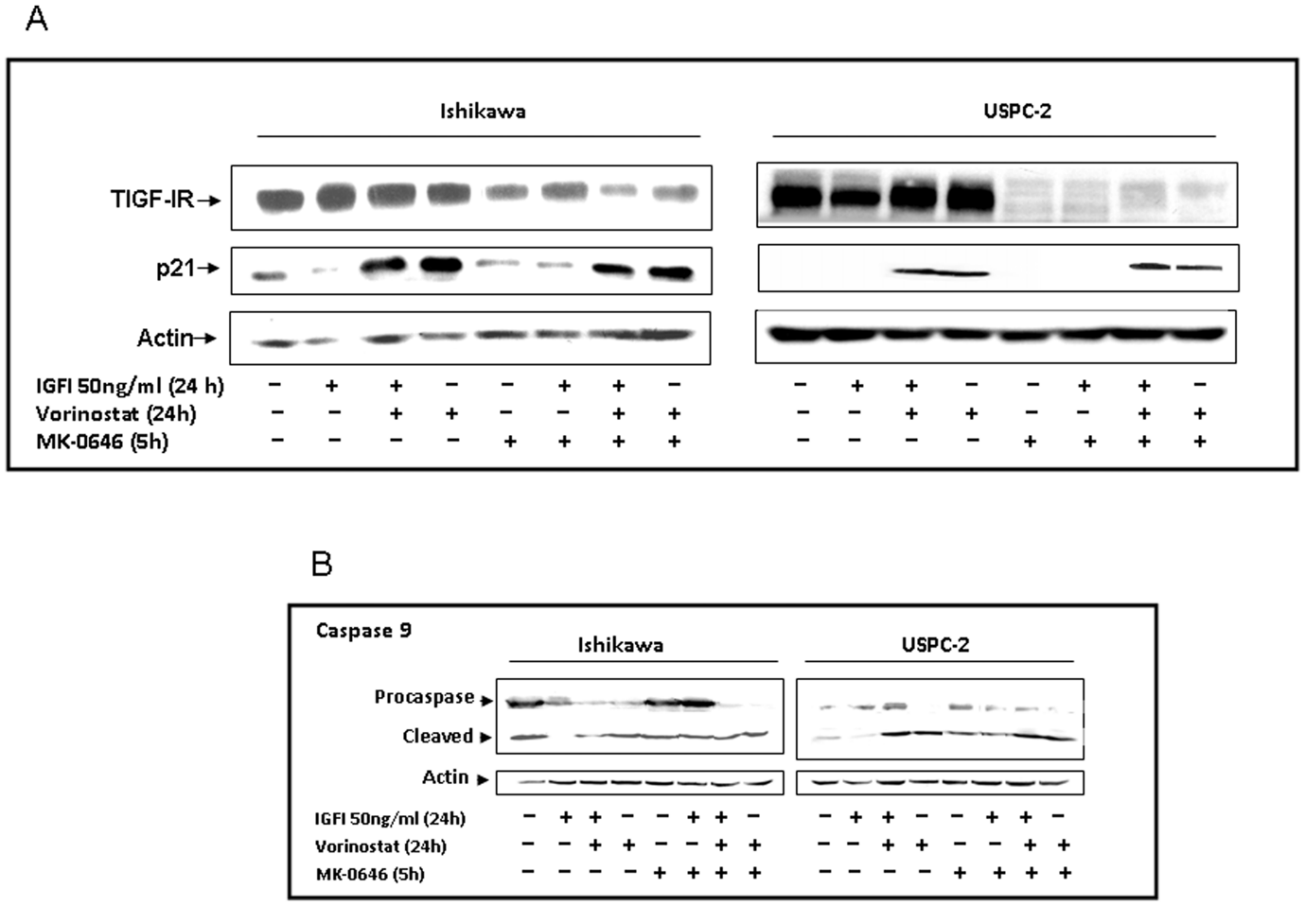
Effect of vorinostat and/or antibody MK-0646 on apoptosis and IGF-IR protein levels. Ishikawa and USPC-2 cells were treated with vorinostat for 24 h, separately and in combination with MK-0646, in the presence or absence of IGF-I. Whole-cell lysates (70 µg for caspase 9 and 100 µg for total IGF-IR and p21 proteins detection) were resolved by SDS-PAGE and immunoblotted with antibodies against T-IGF-IR and p21 (A) and caspase 9 (B). The figure shows the results of a typical experiment, repeated twice with similar results.

Finally, we investigated whether the pro-apoptotic effect of vorinostat requires IGF-IR action. For this purpose, cells were treated with vorinostat for 24 h, separately and in combination with MK-0646, a blocking monoclonal anti-IGF-IR antibody, in the presence or absence of IGF-I, after which apoptosis was assessed by caspase 9 measurements. As shown in Figure 9A, MK-0646, alone and in combination with IGF-I and/or vorinostat, induced a significant decrease in total IGF-IR levels in both cells lines. Furthermore, Western blot analysis revealed that vorinostat abolished the anti-apoptotic activity of IGF-I both in the absence or presence of MK-0646, thus suggesting that the pro-apoptotic action of vorinostat does not require IGF-IR action (Figure 9 B).

## Discussion

Uterine serous carcinoma is a very aggressive cancer for which there are no effective therapeutic protocols. There is, therefore, an urgent need to identify new therapeutic regimens that will prolong survival of USC patients and that will transform response to treatment into cure. In this study we demonstrate that vorinostat, a histone deacetylase inhibitor, markedly induces the accumulation of acetylated histone H3, leading to transcriptional modulation of specific gene programs, including genes involved in IGF-IR and downstream mediators regulation. Our results demonstrate that vorinostat induced apoptosis, growth arrest, and inhibition of migration and colony formation in Type I and II endometrial cancer cell lines. In addition, we demonstrate that inhibition of HDAC modulates IGF-IR expression in Ishikawa and USPC-2 cells lines. The Ishikawa cell line was derived from a well differentiated adenocarcinoma and is a validated model for Type I endometrial cancer. Type I cancers express the estrogen and progesterone receptors [27], exhibit microsatellite instability, and include mutations in the *ras* proto-oncogene, p53 [28] and pTEN tumor suppressor genes, resulting in deregulated PI3K signaling and activation of AKT. In addition, they secrete IL-6, TGF-alpha and IGF-II, but not IGF-I [29]. By contrast, Type II tumors show mutations in p53 [30], but almost never have microsatellite instability or *ras* or pTEN mutations. Usually they overexpress the HER-2/neu oncogene, and secrete IGF's, IGFBP and IL-6.

PTEN was shown to downregulate IGF-II and IGF-IR expression in hepatoma and prostate cancer cells, respectively, suggesting that the anti-proliferative effects of pTEN are, at least in part, mediated through the regulation of expression of components of the IGF system [26], [31]. Vorinostat was shown to down-regulate autocrine IGF-I production and expression, and the IGF-IR in multiple myeloma [32]. A number of studies have shown a correlation between components of the IGF system and endometrial cancer risk. The IGF system has autocrine and paracrine functions in the regulation of endometrial proliferation and differentiation [33]. Results of migration assays suggest that both cell lines secrete growth factors and/or cytokines responsible for their proliferative effects. In particular, a highly mitogenic effect was seen in USPC-2 cells. Vorinostat, both in the presence or absence of IGF-I, inhibited migration of both cell lines. Western blot analyses showed that vorinostat robustly increased the IGF-I-stimulated IGF-IR phosphorylation in Ishikawa cells. Furthermore, alone or in combination with IGF-I, vorinostat up-regulated total IGF-IR levels. A potential explanation for the increase in IGF-IR phosphorylation in Ishikawa cells is the reported vorinostat-induced down-regulation of endogenous IGF-II. This reduction may shift the competition between secreted IGF-II and exogenous IGF-I, leading to an increase in IGF-I binding with ensuing enhanced activation. In addition, the expression of a constitutively-active AKT in Ishikawa cells (as a result of loss of pTEN) significantly increased the total IGF-IR expression. This effect might be mediated at the level of transcription, as shown by the large increase in IGF-IR promoter activity.

A number of epigenetic drugs have already been approved for the treatment of hematological malignancies [34]. These drugs include demethylating agents and HDAC inhibitors such as trichostatin A. Previous studies showed that treatment of mice fibroblasts with trichostatin A induced partial relaxation of genomic imprinting as well as decreased DNA methylation of both IGF-IIR sense and antisense promoters. Hence, increases in histone acetylation can lead to decreased DNA methylation, thereby modulating the regulation of the imprinted expression of IGF-IIR sense and antisense transcripts. Interestingly, Risinger et al reported that promoter hypermethylation is common in Type I, but not Type II, endometrial tumors [35]. HDAC inhibitors can also induce TNF-related apoptosis-inducing ligand (TRAIL) [36], suggesting the activation of the death receptor pathway without the requirement of exogenous TRAIL, leading to activation of caspase 8 [37]. Our data suggest that vorinostat may modulate apoptotic cell death by both the extrinsic and intrinsic pathways in USPC-2 cells because it changed the activation of caspase 8, the expression of Bcl2, and further contributed to caspase 3 activation, following activation of caspase 9. Vorinostat-induced apoptosis in Ishikawa cells might be related to caspases 3 and 9 activation. The activation of caspase 3 was accompanied by PARP cleavage into an 85-kDa fragment in both cells lines. Moreover, our data showed that the pro-apoptotic action of vorinostat is not correlated with IGF-IR levels.

Lu et al reported that a high level of mTor activity is a consistent feature of endometrial carcinomas. Activation of mTor occurred in tumors that had lost pTEN, resulting in deregulated PI3K signaling and activation of AKT. While pTEN alterations are well known in endometrial cancer, signaling defects involving the downstream effector mTor, or other tumor suppressors that regulate mTor signaling, including TSC2 and LKB1, are less documented [38]. Constitutive mTor expression was observed in Ishikawa cells including an inactive pTEN. In addition, vorinostat-induced activation of mTor in USPC-2 cells might be mediated via the PI3K/AKT and p53/Ampk signaling pathways. Alternatively, activation of mTor expression might be induced by p53-independent mechanisms. No data, however, is available regarding the p53 status in USPC-2 cells. Previous investigations have provided evidence to support the view that the presence of an intact p53 is essential for an efficient HDAC inhibitor-induced apoptotic response [39]. This dependence appears to vary with the agent used and may be due to differences in potency. Furthermore, acetylation of p53 occurs following HDAC inhibitor treatment and may increase its activity and reduce targeting of p53 for degradation [40], [41]. However, others have shown that HDAC inhibitors induce apoptosis via p53-independent mechanisms [41]. More experiments are required to define the role of p53 in the vorinostat-mediated apoptosis of USPC-2 cells.

Studies have shown that HDAC inhibitors are relatively non-toxic to normal cells or tissues, exhibiting selective cytotoxicity against a wide range of cancer cells. A number of HDAC inhibitors are currently being investigated in clinical trials against hematological and solid tumors, both as single agents and in combination with other cytotoxic therapies [42]. MTT analysis revealed a significant anti-proliferative activity of vorinostat in both cell lines assayed and a synergistic effect of combined treatment of vorinostat with cisplatin in USPC-2 cells. In addition, cell cycle analyses have shown that vorinostat is able to arrest cell cycle progression in endometrial cancer cells. Recent studies have demonstrated that the HDAC inhibitor-mediated induction of p21 was the result of increased H3 and H4 acetylation associated with the p21 gene promoter [43], [44]. These results are in line with preclinical and clinical data showing that H3 and H4 acetylation are important biomarkers for vorinostat action [18]. P21 is member of the family of endogenous cyclin-dependent kinase inhibitors that negatively regulate cell cycle progression and cyclin D1, a positive regulator of cell cycle progression. Our data show that vorinostat increased p21 expression and inhibited cyclin D1 expression in both cell types, with ensuing growth arrest. In summary, our studies demonstrate that vorinostat exhibits a potent apoptotic and anti-proliferative effect in both Type I and II endometrial cancer cell lines, strongly suggesting that endometrial cancer may be therapeutically targeted by vorinostat.

## References

[1] Bokhman JV (1983). Two pathogenetic types of endometrial carcinoma.. Gynecol Oncol.

[2] Lax SF (2004). Molecular genetic pathways in various types of endometrial carcinoma: from a phenotypical to a molecular-based classification.. Virchows Arch.

[3] Goff BA, Kato D, Schmidt RA, Ek M, Ferry JA (1994). Uterine papillary serous carcinoma: patterns of metastatic spread.. Gynecol Oncol.

[4] Hamilton CA, Cheung MK, Osann K, Chen L, Teng NN (2006). Uterine papillary serous and clear cell carcinomas predict for poorer survival compared to grade 3 endometrioid corpus cancers.. Br J Cancer.

[5] Pallares J, Martinez-Guitarte JL, Dolcet X, Llobet D, Rue M (2004). Abnormalities in the NF-kappaB family and related proteins in endometrial carcinoma.. J Pathol.

[6] Nayak NR, Giudice LC (2003). Comparative biology of the IGF system in endometrium, decidua, and placenta, and clinical implications for foetal growth and implantation disorders.. Placenta.

[7] Gunter MJ, Hoover DR, Yu H, Wassertheil-Smoller S, Manson JE (2008). A prospective evaluation of insulin and insulin-like growth factor-I as risk factors for endometrial cancer.. Cancer Epidemiol Biomarkers Prev.

[8] Werner H, Bruchim I (2009). The insulin-like growth factor-I receptor as an oncogene.. Arch Physiol Biochem.

[9] Werner H, LeRoith D, Zumkeller W, Baxter R (203). The molecular basis of IGF-I receptor gene expression in human cancer.. Insulin-like growth factors.

[10] LeRoith D, Werner H, Beitner-Johnson D, Roberts CT (1995). Molecular and cellular aspects of the insulin-like growth factor I receptor.. Endocrine Rev.

[11] Baserga R, Hongo A, Rubini M, Prisco M, Valentinis B (1997). The IGF-I receptor in cell growth, transformation and apoptosis.. Biochim Biophys Acta.

[12] Gualberto A, Pollak M (2009). Emerging role of insulin-like growth factor receptor inhibitors in oncology: early clinical trial results and future directions.. Oncogene.

[13] Siegel D, Hussein M, Belani C, Robert F, Galanis E (2009). Vorinostat in solid and hematologic malignancies.. J Hematol Oncol.

[14] Kelly WK, Richon VM, O'Connor OA, Curley T, MacGregor-Curtelli B (2003). Phase I clinical trial of histone deacetylase inhibitor: suberoylanilide hydroxamic acid administered intravenously.. Clin Cancer Res.

[15] Kelly WK, O'Connor OA, Krug LM, Chiao JH, Heaney M (2005). Phase I study of an oral histone deacetylase inhibitor, suberoylanilide hydroxamic acid, in patients with advanced cancer.. J Clin Oncol.

[16] Werner H, Bach MA, Stannard B, Roberts CT, LeRoith D (1992). Structural and functional analysis of the insulin-like growth factor I receptor gene promoter.. Mol Endocrinol.

[17] Werner H, Rauscher FJ, Sukhatme VP, Drummond IA, Roberts CT (1994). Transcriptional repression of the insulin-like growth factor I receptor (IGF-I-R) gene by the tumor suppressor WT1 involves binding to sequences both upstream and downstream of the IGF-I-R gene transcription start site.. J Biol Chem.

[18] Brasaemle DL, Attie AD (1988). Microelisa reader quantitation of fixed, stained, solubilized cells in microtitre dishes.. Biotechniques.

[19] Quesnel B, Preudhomme C, Lepelley P, Hetuin D, Vanrumbeke M (1996). Transfer of p16inka/CDKN2 gene in leukaemic cell lines inhibits cell proliferation.. Br J Haematol.

[20] Honda H, Nakamoto T, Sakai R, Hirai H (1999). p130(Cas), an assembling molecule of actin filaments, promotes cell movement, cell migration, and cell spreading in fibroblasts.. Biochem Biophys Res Commun.

[21] Stimson L, La Thangue NB (2009). Biomarkers for predicting clinical responses to HDAC inhibitors.. Cancer Lett.

[22] Duriez PJ, Shah GM (1997). Cleavage of poly(ADP-ribose) polymerase: a sensitive parameter to study cell death.. Biochem Cell Biol.

[23] Wozniak MB, Villuendas R, Bischoff JR, Aparicio CB, Martinez Leal JF (2010). Vorinostat interferes with the signaling transduction pathway of T-cell receptor and synergizes with phosphoinositide-3 kinase inhibitors in cutaneous T-cell lymphoma.. Haematologica.

[24] Marks PA, Breslow R (2007). Dimethyl sulfoxide to vorinostat: development of this histone deacetylase inhibitor as an anticancer drug.. Nat Biotechnol.

[25] Sarfstein R, Maor S, Reizner N, Abramovitch S, Werner H (2006). Transcriptional regulation of the insulin-like growth factor-1 receptor in breast cancer.. Mol Cell Endocrinol.

[26] Zhao H, Dupont J, Yakar S, Karas M, LeRoith D (2004). PTEN inhibits cell proliferation and induces apoptosis by downregulating cell surface IGF-IR expression in prostate cancer cells.. Oncogene.

[27] Hata H, Kuramoto H (1992). Immunocytochemical determination of estrogen and progesterone receptors in human endometrial adenocarcinoma cells (Ishikawa cells).. J Steroid Biochem Mol Biol.

[28] Hollstein M, Sidransky D, Vogelstein B, Harris CC (1991). p53 mutations in human cancers.. Science.

[29] Reynolds RK, Hu C, Baker VV (1998). Transforming growth factor-alpha and insulin-like growth factor-I, but not epidermal growth factor, elicit autocrine stimulation of mitogenesis in endometrial cancer cell lines.. Gynecol Oncol.

[30] Idrees MT, Schlosshauer P, Li G, Burstein DE (2006). GLUT1 and p63 expression in endometrial intraepithelial and uterine serous papillary carcinoma.. Histopathology.

[31] Kang-Park S, Lee YI, Lee YI (2003). PTEN modulates insulin-like growth factor II (IGF-II)-mediated signaling; the protein phosphatase activity of PTEN downregulates IGF-II expression in hepatoma cells.. FEBS Lett.

[32] Mitsiades CS, Mitsiades NS, McMullan CJ, Poulaki V, Shringarpure R (2004). Transcriptional signature of histone deacetylase inhibition in multiple myeloma: biological and clinical implications.. Proc Natl Acad Sci USA.

[33] Rutanen EM (1998). Insulin-like growth factors in endometrial function.. Gynecol Endocrinol.

[34] Esteller M (2007). Epigenetic gene silencing in cancer: the DNA hypermethylome.. Hum Mol Genet.

[35] Risinger JI, Maxwell GL, Berchuck A, Barrett JC (2003). Promoter hypermethylation as an epigenetic component in Type I and II endometrial cancers.. Ann NY Acad Sci.

[36] Fulda S, Debatin KM (2005). HDAC inhibitors: double edge sword for TRAIL cancer therapy?. Cancer Biol Ther.

[37] Ashkenazi A (2002). Targeting death and decoy receptors of the tumour-necrosis factor superfamily.. Nat Rev Cancer.

[38] Lu KH, Wu W, Dave B, Slomovitz BM, Burke TW (2008). Loss of tuberous sclerosis complex-2 function and activation of mammalian target of rapamycin signaling in endometrial carcinoma.. Clin Cancer Res.

[39] Henderson C, Mizzau M, Paroni G, Maestro R, Schneider C (2003). Role of caspases, Bid, and p53 in the apoptotic response triggered by histone deacetylase inhibitors trichostatin-A (TSA) and suberoylanilide hydroxamic acid (SAHA).. J Biol Chem.

[40] Juan LJ, Shia WJ, Chen MH, Yang WM, Seto E (2000). Histone deacetylases specifically down-regulate p53-dependent gene activation.. J Biol Chem.

[41] Ito A, Kawaguchi Y, Lai CH, Kovacs JJ, Higashimoto Y (2002). MDM2-HDAC1-mediated deacetylation of p53 is required for its degradation.. EMBO J.

[42] Gridelli C, Rossi A, Maione P (2008). The potential role of histone deacetylase inhibitors in the treatment of non-small-cell lung cancer.. Crit Rev Oncol Hematol.

[43] Richon VM, Sandhoff TW, Rifkind RA, Marks PA (2000). Histone deacetylase inhibitor selectively induces p21WAF1 expression and gene-associated histone acetylation.. Proc Natl Acad Sci USA.

[44] Okamoto H, Fujioka Y, Takahashi A, Takahashi T, Taniguchi T (2006). Trichostatin A, an inhibitor of histone deacetylase, inhibits smooth muscle cell proliferation via induction of p21(WAF1).. J Atheroscler Thromb.

